# OnabotulinumtoxinA for chronic migraine: efficacy, safety, and tolerability in patients who received all 5 treatment cycles in PREEMPT

**DOI:** 10.1186/1129-2377-14-S1-P199

**Published:** 2013-02-21

**Authors:** SK Aurora, DW Dodick, RE DeGryse, CC Turkel

**Affiliations:** 1 Swedish Neuroscience Institute, Seattle, WA, USA; 2 Mayo Clinic Arizona, Dept. of Neurology, Phoenix, AZ, USA; 3 Allergan, Irvine, CA, United States, USA; 4 Allergan, Irvine, CA, United States, UK

## Introduction

Chronic migraine (CM) is a prevalent, disabling neurological disorder. PREEMPT demonstrated efficacy & safety of onabotulinumtoxinA (BOTOX®) for prophylaxis of headaches in adults with CM.

## Objective

Assess patients who received all 5 treatment cycles and completed PREEMPT clinical program.

## Methods

PREEMPT (two phase 3 studies: 24-wk, double-blind, placebo-controlled [DBPC], parallel-group phase, followed by 32-wk, open-label phase) evaluated efficacy & safety of onabotulinumtoxinA in CM (≥15 days/month with headache lasting ≥4 hr/day). Patients were randomized (1:1) to onabotulinumtoxinA (O) or placebo (P) every 12 wks for 2 cycles, followed by onabotulinumtoxinA for 3 cycles. Multiple headache symptom measures were evaluated. Results for the completer (5 cycles) subgroup of patients are reported.

## Results

Of 1384 total patients, 1005 received all 5 treatment cycles [513 received O only (O/O); 492 received 2 cycles of P then 3 cycles of O (P/O)]. Demographics were similar between treatment groups. At Week 56, after all patients were treated with onabotulinumtoxinA, there continued to be significant between-group differences favoring O/O vs P/O group for the following headache symptom measures: mean change from baseline in frequencies of headache days (-12.0 O/O, -11.1 P/O;p=0.035), migraine days (-11.6 O/O, -10.7 P/O;p=0.038), moderate/severe headache days (-11.0 O/O, -10.1 P/O;p=0.042). Treatment-related adverse event rate was 28.5% for O vs 12.4% for P in the DBPC phase and 34.8% for patients treated with O for all 5 cycles throughout the 56-wk trials.

## Conclusion/relevance

This subgroup analysis demonstrated statistically & clinically meaningful improvements with onabotulinumtoxinA treatment (5 cycles) vs placebo (2 cycles)/onabotulinumtoxinA (3 cycles) for multiple headache symptom measures & suggests that at Week 56, patients treated earlier with onabotulinumtoxinA had better outcomes. OnabotulinumtoxinA is an effective, safe, well-tolerated long-term (3-5 cycles) treatment for prophylaxis of headache in adults with CM.

## Support

Allergan, Inc.

